# Physical Activity and Lung Function Growth in a Cohort of Chinese School Children: A Prospective Study

**DOI:** 10.1371/journal.pone.0066098

**Published:** 2013-06-19

**Authors:** Jie Ji, Su-qing Wang, Yu-jian Liu, Qi-qiang He

**Affiliations:** School of Public Health/Global Health Institute, Wuhan University, Wuhan, Hubei Province, P. R. China; University of Southampton, United Kingdom

## Abstract

**Backgrounds/objectives:**

Evidence on the association between physical activity and lung function in children is sparse. The aim of this study was to evaluate children’s lung function growth in relation to their physical activity level in Chinese children.

**Methods:**

A total of 1713 school children aged 9.89±0.86 years who were asthma-free at baseline were followed-up for 18 months from 2006 to 2008 in Guangzhou, China. Information on physical activity and other socio-economic status were obtained from self-administered questionnaires. Lung function tests were performed with a standard procedure.

**Results:**

At the baseline survey, physically active girls had significantly higher forced vital capacity (FVC) than inactive girls (1.79 l vs. 1.75 l, p<0.05). The growth rates for lung function indices were significantly higher for girls who were physically active at either or both follow-up surveys than those inactive at both surveys during the follow-up period forced expiratory flows at 25% (FEF_25_) difference per year (dpy) (0.20 l/s vs. 0.15 l/s), forced expiratory flows at 75% (FEF_75_) dpy (0.57 l/s vs. 0.45 l/s) and forced expiratory flows between 25% and 75% (FEF_25-75_) dpy (0.36 l/s vs. 0.28 l/s) (all p<0.05).

**Conclusions:**

Physical activity is positively associated with lung function growth among Chinese school-aged girls. Promotion of physical activity among children is of great importance.

## Introduction

There is a global trend of decreasing levels of physical activity across all ages in the last few decades. In particular, children have been found to be engaging less physical activity than before. An updated report combining data from 105 countries showed that 80.3% children aged 13 to 15-year-old did not achieve recommended level of physical activity [1], [2].

Physical activity is known to improve muscle strength, enhance bone accretion, prevent child obesity and reduce the risk of cardiovascular diseases [3]. Most previous studies on the effect of physical activity on lung function were conducted in adults [4]–[6]. However, although impaired lung function in childhood may persist into adults [7], there is a paucity of evidence concerning children; and they were limited by the cross-sectional design, and produced equivocal results [8]–[10].

China has been experiencing enormous economic transformations for more than 30 years, while socio-economic status has been shown to be an important determinant of health and physical activity [11], [12]. With the popularity of computer and internet activities, children spend more time being sedentary, and less time in physical activity than before. A recent Chinese national survey revealed that only 22.7% of Chinese students aged 9–18 years met the recommended intensity of physical activity [13]. As no published studies have assessed the association between physical activity and lung function growth in Chinese children, we analyzed the data collected from a prospective study in Guangzhou, China to examine the effect of physical activity on the growth rates in pulmonary function among Chinese school-aged children.

## Methods

### Subjects and Design

The data were obtained from a prospective cohort study on air pollution and school children’s respiratory health in Guangzhou, China. Details of the study design and methods have been described previously [14]. Briefly, one to four primary schools were selected based on their proximity to the air monitoring stations and the required sample size. Students in Grade 3 and 4 in the selected schools were recruited to participate in the study. The baseline and follow-up study were conducted from September to November 2006 and from March to May 2008, respectively. Written informed consents were obtained from all parents of participating children prior to the study. The study has been approved by the Medical Research Ethics Committee of Sun Yat-sen University.

### Physical Activity Assessment

In each survey, before the lung function tests, habitual physical activity among the school children was assessed using a self-administered questionnaire completed in the classroom with the guidance of trained investigators. The questionnaire included the frequency and duration of indoor/outdoor sports (ballgames, running/jogging, track-and-field sports, swimming, dancing, etc.) and free play during the previous year. Based on the children’s responses, their physical activity status was dichotomized into physically active and inactive. Physically active children were defined as those who participated in sports and/or vigorous free play at least three times per week for at least 30 min each time.

### Lung Function Test

In each phase of the study, spirometric tests were conducted in the school by the same group of trained technicians using a Minato AS-505 portable electric spirometer (Minato Ltd, Tokyo, Japan) according to the standards by Miller et al [15]. A 2-L syringe was used to calibrate the spirometer before and after the test on each day. Pulmonary function measurements included: forced vital capacity (FVC), forced expiratory volume in 1 s (FEV_1_), forced expiratory flows at 25% (FEF_25_), 75% (FEF_75_) and between 25% and 75% (FEF_25–75_) of vital capacity. All measurements were performed in a standing position with a nose clip. Each subject was asked to perform the test until three satisfactory measurements were obtained. The average of the three trials was taken as the value for each measurement.

### Other Measurements

Children’s height (standing erect without shoes) and weight (in light clothes) were measured by the same group of technicians using standard methods in each survey. Body mass index (BMI) was calculated by dividing weight (kg) by height squared (m^2^). Information of maternal education and exposure to passive smoke in the home were reported by their parents using a self-administered questionnaire.

### Statistical Analysis

We annualized the growth rates of all anthropometric and lung function indices for each child by calculating the changes in height, weight, BMI and lung function parameters between the baseline and the follow-up measurement. The results were presented as “difference per year” (dpy) for each corresponding index measured.

Results were stratified by sex because we found significant interaction effects between physical activity and gender in the primary analysis. Chi-square(χ^2^) test was used to compare the differences in personal characteristics between physically active and inactive children at baseline. Differences in the anthropometric and lung function indices in each survey, as well as the growth rates of these parameters between physically active and inactive children were analyzed by t-tests and analysis of covariance (ANCOVA) when appropriate. In the cross-sectional data analysis, lung function was adjusted for district, age, height, BMI, passive smoking, and maternal education. In the longitudinal data analysis, the growth rate of lung functions was adjusted for district, age in 2008, height in 2006, change in height between 2006 and 2008, BMI in 2006, change in BMI between 2006 and 2008, lung function indices in 2006, passive smoking, and maternal education. All statistical analyses were performed using the SPSS statistical package (version 13.0; SPSS, Inc., Chicago, IL).

## Results

In the baseline study, a total of 2407 children performed the spirometry tests, and 2179 (90.5%) accomplished acceptable and reproducible tests (on three attempts). In the present study, we included a total of 1713 children (858 boys and 855 girls) aged 9–11 years (mean age = 9.89, SD = 0.86) who completed the follow-up study and were free of asthma at the baseline. Compared to 466 children who did not participate in the follow-up survey (lost to follow-up or excluded from the study), the participant children had similar personal characteristics, including age, anthropometric characteristics, and spirometric indices.

Table 1 shows the baseline characteristics of study subjects stratified by physical activity and sex. No significant differences were observed between physically active and inactive children, except that more physically inactive children were exposed to household passive smoking compared to active ones.

**Table 1:** Baseline characteristics of study subjects.

	Boys			Girls		
	PA(n = 362)	PI(n = 496)	P value	PA(n = 283)	PI(n = 572)	P value
Age, years	10.2(0.8)	10.0(0.9)	0.003	10.0(0.8)	10.0(0.8)	0.472
Height, cm	138.4(6.8)	137.5(6.6)	0.051	139.1(7.7)	138.9(7.4)	0.711
Weight, kg	34.0(8.0)	33.4(8.1)	0.313	32.2(7.0)	32.3(7.2)	0.815
BMI, kg/m^2^	17.6(3.1)	17.5(3.3)	0.864	16.5(2.5)	16.6(2.6)	0.566
Maternal education-low*	27(7.7)	34(6.4)	0.470	20(7.4)	38(7.0)	0.820
Passive smoking household*	57(13.8)	67(16.1)	0.372	21(7.5)	72(12.9)	0.018

Values are presented as mean (SD) or number (percentage) when appropriate.

PA: physical active; PI: physical inactive.

Maternal education-low: ≤6 year education.

* there were some missing values.

Table 2 shows the differences of lung function between physically active and inactive children by sex in baseline and follow-up surveys. Compared to inactive girls, physically active girls had significantly higher FVC (1.79 l vs. 1.75 l, p<0.05) in the baseline survey and insignificantly higher lung function parameters in the follow-up study.

**Table 2:** Differences of lung function between PA and PI children in the study.

	n	FVC	FEV_1_	FEF_25–75_	FEF_75_	FEF_25_
**Baseline survey**						
Boys						
PA	362	1.93(0.01)	1.74(0.01)	2.08(0.03)	2.97(0.05)	1.28(0.02)
PI	496	1.92(0.01)	1.73(0.01)	2.14(0.03)	2.92(0.04)	1.33(0.02)
Girls						
PA	283	1.79(0.02)	1.62(0.02)	2.05(0.04)	2.60(0.06)	1.36(0.03)
PI	572	1.75(0.01)*	1.59(0.01)	2.06(0.03)	2.59(0.04)	1.37(0.02)
**Follow-up survey**						
Boys						
PA	393	2.39(0.02)	2.12(0.01)	2.50(0.03)	3.62(0.05)	1.51(0.03)
PI	465	2.37(0.01)	2.11(0.01)	2.55(0.03)	3.60(0.05)	1.54(0.02)
Girls						
PA	297	2.21(0.02)	2.02(0.02)	2.63(0.04)	3.47(0.06)	1.70(0.03)
PI	558	2.19(0.01)	2.01(0.01)	2.55(0.03)	3.39(0.05)	1.64(0.02)

Data are presented as mean (SE), after adjustment for district, age, height, BMI, passive smoking, and maternal education.

PA: physical active; PI: physical inactive.

* : p<0.05.

Table 3 displays the differences in the annual lung function growth rates between physically active and inactive children. Compared with those physically inactive at both baseline and follow-up surveys, the girls who were physically active at either or both surveys had significantly higher FEF_25_ dpy (0.20 l/s vs. 0.15 l/s), FEF_75_ dpy (0.57 l/s vs. 0.45 l/s) and FEF_25–75_ dpy (0.36 l/s vs. 0.28 l/s) during the follow-up period (all p<0.05).

**Table 3:** Differences in the annual growth rates of lung function# between active and inactive children.

	Boys				Girls			
	Active (n = 535)		Inactive (n = 323)		Active (n = 452)		Inactive(n = 403)	
	Mean	SE	Mean	SE	Mean	SE	Mean	SE
FVC dpy, l	0.29	0.01	0.29	0.01	0.28	0.01	0.27	0.01
FEV_1_ dpy, l	0.24	0.01	0.25	0.01	0.27	0.01	0.25	0.01
FEF_25–75_ dpy, l/s	0.26	0.02	0.26	0.02	0.36	0.02	0.28*	0.02
FEF_75_ dpy, l/s	0.44	0.03	0.38	0.04	0.57	0.03	0.45*	0.04
FEF_25_ dpy, l/s	0.13	0.01	0.14	0.02	0.20	0.02	0.15*	0.02

Active: physically active at either survey or both surveys; Inactive: physically inactive at both surveys.

dpy:difference per year;

# Adjusted for district, age in 2008, height in 2006, change in height between 2006 and 2008, BMI in 2006, change in BMI between 2006 and 2008, lung function indices in 2006, passive smoking, and maternal education.

* p<0.05.

## Discussion

The results of the present study suggest that there is a significant association between physical activity and lung function growth among Chinese girls aged 9–11 years. Physically active girls had significantly higher lung function and growth rates in lung function indices than physically inactive girls during an 18-month follow-up period.

Our findings are in accordance with previous studies conducted on adults. In Norway, Nystad et al. found a trend of decline in lung function with decreasing level of physical activity in all age groups in both men and women [4]. Another two longitudinal studies demonstrated that physical activity was associated with a slower rate of decline in pulmonary function during 3.7-year and 25-year follow-up period [5], [6], respectively. However, the findings from adults may not always be extrapolated to children because they have higher elastic recoil than adults and the elastic recoil decreases with age. Aging process results in a decline in lung function in adults [5], while children are at the stage of lung function growth. Additionally, physical activity intensity and physiology are significantly different between adults and children. Although a cross-sectional study in Québec youth found no association between habitual physical activity and pulmonary function both in childhood and adolescent [8], results from our prospective cohort study are consistent to another two previous cross-sectional studies conducted in children. In analyzing the data from 2357 Norwegian children aged 9–10 years, Berntsen and colleagues found that FVC and FEV_1_ tended to increase with increasing level of physical activity when adjusting for potential confounders [9]. In another study with a small sample size (55 Chinese children aged 5–10 years), Jones et al. reported that physical active children had a greater ventilatory capacity than inactive peers [10].

The significant deficits in lung function growth were found only in physically inactive girls in our study. There is no consensus on the gender-specific effects of physical activity on childhood lung function. Similarly to our results, Berntsen et al. found that the association between lung function and physical activity was only present among girls (*p = *.01) [9]. The reason was that tall boys were more likely to attend sports, while girls were not the same. Thus, height reduced the effect of physical activity on lung function among boys but not in girls. In contrast, a cohort study displayed that men who remained active for 5 years had significantly higher mean changes in lung function indices than the other groups, nevertheless, it was not the case for women [16]. The gender difference might be explained by the fact that women had higher lung efficiency and men had more reserve capacity. However, some studies did not demonstrate such findings, and no gender-specific were found [4], [6]. In the present study, lung function and lung function growth have been adjusted for anthropometric characteristics such as height and BMI, etc., thus bias induced by height is impossible. Boys are more active than girls [1], [17], differences of physical activity intensity between active and inactive boys may not be large enough to induce significant differences in lung function growth compared with girls.

In the current study, the deficits in lung function growth for inactive girls were observed only in FEF but not FEV_1_ and FVC. In clinical practice, the FVC measurement is used mostly for the evaluation of large-airway functions; FEV_1_ reflects airway obstruction in both large and small airways, whereas FEF_25_, FEF_75_, and FEF_25–75_ reflect peripheral small-airway functions. Laura Seed et al. [18] concluded in their review that FEF_25–75_ is the best indicator of early small airway dysfunction in children, and is more sensitive than the FEV_1_. Similarly, Wetter et al. chose small airway reactivity and resistance as outcome variables. They found that the test of large airway resistance showed no effect of maximum exercise [19]. Therefore, the possibility that physical activity may lead to preclinical structural changes on small airways before the larger airways warranted further investigation.

There is some uncertainty on the mechanism of effects of physical activity on respiratory function. Donaldson et al. reported that exercise interventions during or shortly after an exacerbation of COPD requiring hospitalization resulted in significant improvements in quadriceps muscle strength [20]. On the other hand, Rosenkranz et al. found that in non-asthmatic prepubescent children, inactivity negatively impacted airway responsiveness, which may be improved with high-intensity training [21]. Furthermore, physical activity might affect pulmonary function through an effect on obesity. For instance, Rupert W. Jakes observed an association between physical inactivity and obesity and fat distribution [6]. Thus, physical activity may affect lung function both directly and indirectly.

Strengths of this study include the longitudinal design, the large sample size and a high follow-up rate. The participants’ characteristics were broadly similar to those lost to follow-up. Moreover, the spirometric tests in both the baseline and follow-up study were conducted by the same group of technicians.

Caution is necessary when interpreting the results because of the following limitations. Firstly, the measure of physical activity was based on the children’s self-reported questionnaire, which might have been influenced by the ability to accurately recall. Estimated physical activity levels have been shown to be consistently higher than those measured by other instruments [22]. Although the test-retest reliability study showed a good intra-class correlation coefficient (r = 0.78), the questionnaire has not been validated in the schoolchildren. Thus, this is a limitation in this study. However, direct determinations of physical activity level are impractical in population-based studies. Furthermore, it is currently the most commonly used method in epidemiological studies. Secondly, although several important and potentially confounding factors have been adjusted in the statistical analyses, other confounding factors, such as dietary energy intake information and pubertal level, which have not been assessed, could have influenced the results, thus some unmeasured confounding effects might exist. Finally, baseline lung function was conducted from September to November, but the follow-up was conducted from March to May. Therefore, some of the differences could be due to seasonal differences.

In conclusion, the present prospective study documents a significant positive relationship of physical activity with lung function growth in Chinese girls. The results of the current study underline the importance of physical activity promotion in school-aged children.
